# Uterine Sandwich Suture: The Concept of “Role Sharing” Is Important

**DOI:** 10.7759/cureus.11492

**Published:** 2020-11-15

**Authors:** Shigeki Matsubara, Hironori Takahashi

**Affiliations:** 1 Obstetrics and Gynecology, Jichi Medical University, Shimotsuke, JPN

**Keywords:** bakri balloon, b-lynch suture, obstetric hemorrhage, uterine compression suture, uterine sandwich

## Abstract

Uterine compression suture and intrauterine hemostatic balloon are important procedures to achieve hemostasis for obstetric hemorrhage. A combined use of these two, with B-Lynch suture + Bakri balloon being the most often employed ones, is referred to as a “uterine sandwich”, which is an effective hemostatic procedure. Fundamentally, the former and latter stop bleeding from the uterine body and lower uterine segment, respectively. This represents the concept of “role sharing” for hemostasis. Recognizing this concept is of practical importance.

## Introduction

A recent article by Farshchian and Castaneda in Cureus dealt with “uterine sandwich” for obstetric hemorrhage [1]: we welcome this article, which may further widen the use of this technique worldwide. In a patient with obstetric hemorrhage at cesarean section (CS), “uterine sandwich”, B-Lynch uterine compression suture (UCS) [2] + Bakri balloon [3], was employed, which achieved hemostasis. This patient had velamentous cord insertion (VCI) and posterior placenta previa [1]. We wish to explain some important issues regarding the uterine sandwich and thereby re-emphasize its fundamental concept, which may be clinically useful.

## Technical report

A uterine sandwich can be used for obstetric hemorrhage at both vaginal and cesarean delivery. In the former, performing a uterine sandwich requires a new laparotomy, whereas, in the latter, it can be performed during CS because bleeding usually occurs during CS. For simplicity, we wish to describe the latter case. 

A Bakri balloon should be inserted from the hysterotomy window during CS [4]. This balloon is usually placed in the lower uterine segment (the lower part of the uterus). Then, with care not to puncture the intrauterine balloon, B-Lynch UCS should be placed. On confirming hemostasis for hemorrhage from both the upper and lower uterine parts, hysterotomy should be closed. In the hysterotomy closure, care should also be taken to avoid puncturing the balloon. As will be described later, UCS other than B-Lynch (for example, Matsubara-Yano [MY] suture [5,6] or Hayman UCS [7]) can be used for a uterine sandwich, in which case hysterotomy should be closed before performing UCS. 

**Figure 1:**
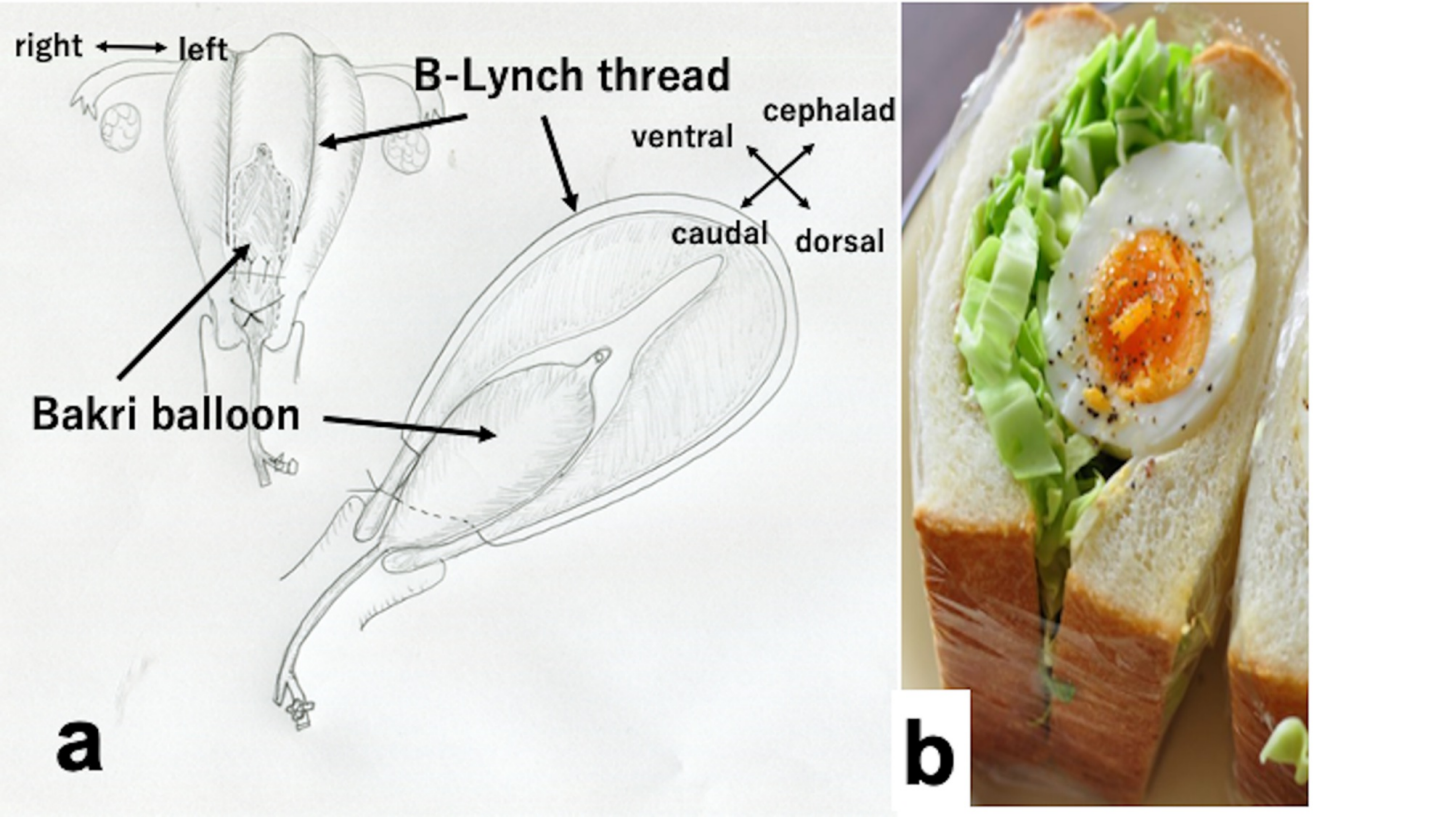
Schematic explanation of uterine sandwich (a) and sandwich (b). (a) A Bakri balloon is inserted and placed around the lower uterine segment. An intrauterine balloon usually tends to descend caudally (balloon prolapse into the vagina). B-Lynch uterine compression suture is placed, compressing the uterus, and thereby also fixing the Bakri balloon intrauterine; this also prevents the balloon from prolapsing. Although some uterine compression sutures are designed to achieve hemostasis of the lower uterine segment, many (including B-Lynch) are designed to achieve hemostasis of the uterine body (uterine upper part). Thus, the fundamental concept of the uterine sandwich is as follows: B-Lynch suture for bleeding from the uterine body, and Bakri balloon for bleeding from the lower uterine segment. This is the concept of “role sharing”. (b) An egg sandwich. The boiled egg represents Bakri balloon. Instead of B-Lynch suture (thread), the sandwich is wrapped in plastic, which holds the sandwich shape.

The concept of a uterine sandwich is as follows: most types of UCS (including B-Lynch, Hayman, and MY sutures) were designed to stop bleeding from the uterine body [2,5-7]. This is evident from the figure in Farshchian and Castaneda’s study [1] and also our Figure 1a: a thread fundamentally compresses the uterine body and not the lower uterine segment (site that previa placenta adheres to). Conversely, a Bakri balloon fundamentally compresses the lower uterine segment (at least lower half of the uterus), stopping the hemorrhage (Figure 1a) [3,8]. Full inflation of the Bakri balloon (500 mL), in some cases, is attempted to stop bleeding also from the uterine body (upper part); however, a Bakri balloon primarily stops the bleeding from the lower segment [3,8]. B-Lynch suture is for bleeding from the upper part [2,5-7], and a Bakri balloon is for bleeding from the lower part [3,8].

Figure 1 illustrates the schema of the uterine sandwich. The boiled egg represents Bakri balloon and sliced bread represents the uterine wall; B-Lynch suture, compressing the uterus (thus bread), fixes egg + bread, maintaining the sandwich. The uterine sandwich, compressing from both inside and outside the uterus, stops bleeding from both the upper and lower uterine part.

## Discussion

We briefly summarized some technical points and, more importantly, the fundamental concept of the uterine sandwich. The concept of “role sharing” is important.

Briefly reviewing the history of hemostasis for obstetric hemorrhage, obstetric hysterectomy has long been employed in patients with severe hemorrhage that does not respond to conventional treatments, such as uterine massage, bimanual uterine-compression, and uterotonic administration. However, obstetric hysterectomy is technically difficult and results in loss of fertility [9]. Recently, several hemostatic procedures have been introduced and are widely acknowledged, which preserve the uterus. Of them, UCS and the intrauterine hemostatic balloon have markedly changed the daily practice for obstetric hemorrhage. B-Lynch UCS [2,6] and Bakri balloon [3] represent the former and latter, respectively. We believe that these two procedures are epoch-making in obstetric history: Doctors B-Lynch and Bakri made gigantic steps forward in medicine.

To our knowledge, Nelson and O’Brien [10] were the first to combine these two procedures (B-Lynch suture and a Bakri balloon), introducing the standard uterine sandwich. Some modifications to this uterine sandwich (B-Lynch + Bakri) have been made. We also devised the MY sandwich, which effectively stops hemorrhage [11,12]: MY UCS was used instead of B-Lynch UCS [5,6]. As previously described [5,6,11,12], MY UCS overcame some drawbacks of B-Lynch UCS, and thus the MY sandwich also overcame those of the conventional uterine sandwich (B-Lynch + Bakri). We previously explained how such modifications may function [11,12] and will avoid repetitive explanation. The important point is the concept of uterine sandwich, “role sharing”. This concept is common to any modified uterine sandwich procedures.

In many cases, obstetric uterine hemorrhage occurs in both upper and lower uterine parts. We wish to explain the situation with two conditions as examples. Atonic bleeding usually occurs from the uterine body, and bleeding at placenta previa usually occurs from the lower uterine segment (main attachment sites of previa placenta). However, the situation in real-world obstetric practice is not so straightforward. Uterine atony sometimes involves not only the uterine body but also the lower segment. In placenta previa, the placenta sometimes extends from the lower segment to the uterine body. Under these conditions, bleeding occurs both from the uterine body and lower segment, sometimes at the same time and sometimes with some time lag. Not confined to these two conditions, we often encounter patients in whom bleeding occurs from both upper and lower uterine parts [11,12]. We must stop bleeding from both. A uterine sandwich, a hemostatic procedure with “role sharing”, may be effective in these situations.

We wish to make additional comments on Farshchian and Castaneda’s case, thereby further highlighting the clinical significance of the uterine sandwich. The patient had VCI and “posterior” placenta previa: the authors overly emphasized the presence of these two conditions, which is misleading. In VCI, when a large cord vessel runs on/around the internal ostium, which is referred to as vasa previa, vaginal delivery often injures the cord vessels and causes life-threatening fetal hemorrhage, and thus CS should be performed. At CS, hysterotomy at too near close to the cord may injure the cord vessels, also causing marked fetal hemorrhage, and thus hysterotomy should be conducted away from the cord or cord vessels. With these cautions, however, VCI itself does not require a uterine sandwich. This patient had “posterior” placenta previa, and actually a uterine sandwich was a good treatment choice. However, a uterine sandwich was meaningful not because previa was located “posteriorly” but because hemorrhage occurred during CS. A uterine sandwich is not applicable for some specific conditions, i.e., VCI or “posterior” placenta previa, but it is applicable for obstetric hemorrhage in general when bleeding occurs or is expected to occur from both upper and lower parts of the uterus.

## Conclusions

In the uterine sandwich, the concept of role sharing is important. UCS is performed for the bleeding from the uterine upper part, and Bakri balloon is for bleeding from the uterine lower part. Understanding this may be especially important in an emergency situation. One may forget the technical details of the uterine sandwich; however, understanding this fundamental concept may help physicians, especially less-experienced physicians, handle life-threatening obstetric hemorrhage. The process leading to the establishment of Hayman suture showed that understanding the concept, and not simply focusing on the technique itself, led to this innovative UCS.
